# A rapid and sensitive system for recovery of nucleic acids from *Mycobacteria sp.* on archived glass slides

**DOI:** 10.1186/s12866-018-1335-0

**Published:** 2018-11-26

**Authors:** Balkis A. Talip, William J. Snelling, Roy D. Sleator, Colm Lowery, James S. G. Dooley

**Affiliations:** 1 School of Biomedical Sciences, Faculty of Life and Health Sciences, Ulster University, Cromore Road, BT52 1SA Coleraine, County Londonderry Northern Ireland; 2 Faculty of Applied Sciences and Technology, Universiti Tun Hussein Onn Malaysia, 84000 Pagoh, Muar, Johor Malaysia; 3 Department of Biological Sciences, Cork Institute of Technology, Bishopstown, Cork, Ireland

**Keywords:** Laser capture microscope, Multiple displacement amplification, Nested real-time PCR

## Abstract

**Background:**

The field of diagnostics continues to advance rapidly with a variety of novel approaches, mainly dependent upon high technology platforms. Nonetheless much diagnosis, particularly in developing countries, still relies upon traditional methods such as microscopy. Biological material, particularly nucleic acids, on archived glass slides is a potential source of useful information both for diagnostic and epidemiological purposes. There are significant challenges faced when examining archived samples in order that an adequate amount of amplifiable DNA can be obtained. Herein, we describe a model system to detect low numbers of bacterial cells isolated from glass slides using (laser capture microscopy) LCM coupled with PCR amplification of a suitable target.

**Results:**

*Mycobacterium smegmatis* was used as a model organism to provide a proof of principle for a method to recover bacteria from a stained sample on a glass slide using a laser capture system. Ziehl-Neelsen (ZN) stained cells were excised and catapulted into tubes. Recovered cells were subjected to DNA extraction and pre-amplified with multiple displacement amplification (MDA). This system allowed a minimum of 30 catapulted cells to be detected following a nested real-time PCR assay, using *rpoB* specific primers. The combination of MDA and nested real-time PCR resulted in a 30-fold increase in sensitivity for the detection of low numbers of cells isolated using LCM.

**Conclusions:**

This study highlights the potential of LCM coupled with MDA as a tool to improve the recovery of amplifiable nucleic acids from archived glass slides. The inclusion of the MDA step was essential to enable downstream amplification. This platform should be broadly applicable to a variety of diagnostic applications and we have used it as a proof of principle with a *Mycobacterium* sp. model system.

## Background

Despite significant advancements in recent years, Tuberculosis (TB) remains a global public health concern [1]. Indeed, one third of the world’s population is infected with TB, with 9.6 million people worldwide became sick with the disease in 2014 (http://www.cdc.gov/tb/statistics/). Additionally, the emergence of infectious non-tuberculous mycobacteria (NTM) has become a problem of increasing clinical significance. Over the past 20 years, numerous cases involving NTM have been reported worldwide [2].

Appropriate surveillance and detection systems are the first line of defence in the global war on TB [3]. In high-TB-burden countries such as Africa, India, and several South East Asian countries, diagnosis is typically by means of sputum analysis [4]. This is a two-step process involving an initial screen for acid-fast bacilli by microscopy (Ziehl-Neelsen stain), followed by bacterial culture [5]. Although rapid, conventional microscopy based detection methods, lack sensitivity. Bacterial culture, on the other hand, while more sensitive, is also more time consuming, requiring up to 12 weeks for confirmation. With the emergence of multi-drug resistant TB (MDR-TB) and non-tuberculous mycobacteria (NTM), both involved in human disease, the development of more rapid and effective detection systems is urgently required [6, 7]. To this end, several studies have indicated the potential of applying molecular detection systems to infected tissue/sputum samples, fixed on pre-stained glass slides as an alternative to culture [8–10]. Although this approach facilitates rapid diagnosis, a number of significant limitations exist; not least of which are the myriad inhibitory factors from the adjacent tissue which is likely to seriously compromise assay sensitivity. In addition, the numbers of cells found on the slide is typically low, particularly from sputum samples, often leading to misinterpretation of results [10]. Several studies have shown that multiple displacement amplification (MDA) can be used as the pre-polymerase chain reaction to process minute amounts of DNA [11, 12]. MDA has also been used to process archived/ancient samples [13] and within tissues/membrane [14–19]. These reports and others have set the stage for using MDA to develop a rapid diagnostic system for TB and NTM, especially for samples treated under harsh conditions, such as Ziehl-Neelsen (ZN) staining. Furthermore, this approach is likely to be exquisitely sensitive, given that MDA can be reliably applied to a single bacterial cell [19–22]. Employing the phage Φ 29 DNA polymerase, which exhibits robust polymerization activity and high enzymatic fidelity; MDA is an ideal pre-PCR procedure for the detection of low copy number sequences [17]. Herein, using *Mycobacterium smegmatis* as a model organism, we describe the use of MDA in combination with LCM for the development of a sensitive and rapid diagnostic platform for targeting TB. While previous studies have indicated the use of Whole genome amplification (WGA) as a pre-PCR system to increase the sensitivity of detection for minute quantities of starting material, ours is the first to report the use of MDA for the detection of single bacterial cells isolated from pre-stained archived glass slides, using LCM. Specifically, we describe a new approach to increase the sensitivity of detection of *M. smegmatis* isolated from archived glass slides using MDA followed by PCR and real-time nested PCR. In addition, by comparing three different DNA extraction methods we highlight the most suitable method used to extract DNA from low numbers of cells catapulted using LCM.

## Results

### Isolation of a single cell of *M. smegmatis* from Ziehl-Neelsen (ZN) stained archived slides using laser capture microscope (LCM)

**Fig. 1:**
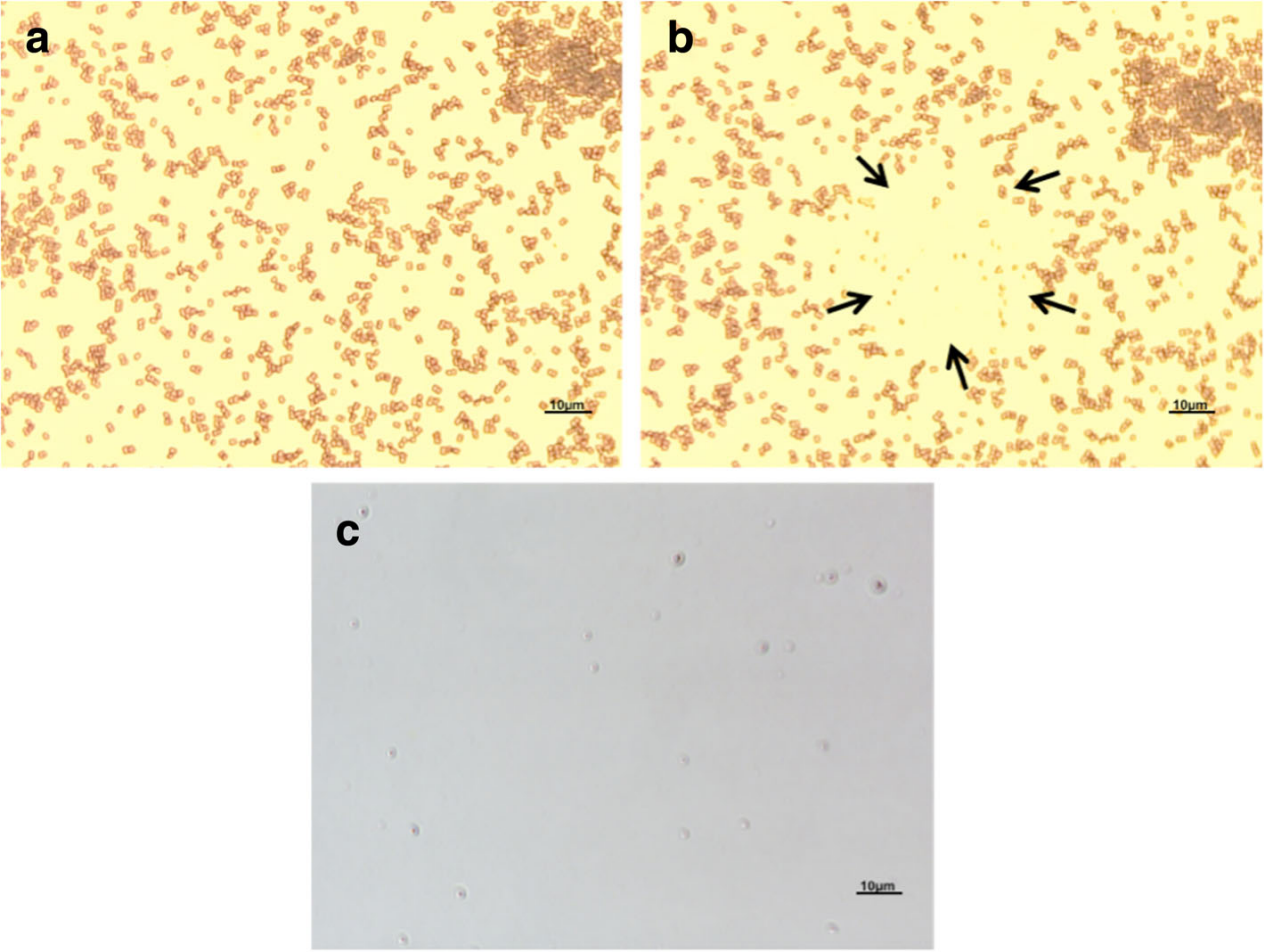
**a-c**: The *M. smegmatis* ZN stained slides observed and isolated from glass slides using 63× magnification of LCM. **a** ZN stained slides before catapulting **b** The selected area was catapulted by using LPC application **c** Post-catapulted materials (PCM) were re-examined to ensure the cells were successfully collected

The ZN stained *M. smegmatis* (48 h old culture) under the LCM exhibits a “pale-red” colour due to its acid-fast property (Fig. 1 a and b). The LPC application of LCM was used to perform the isolation process and confirm that it could be used as a potential method to isolate a single bacterial cell fixed on a glass slide.

**Fig. 2:**
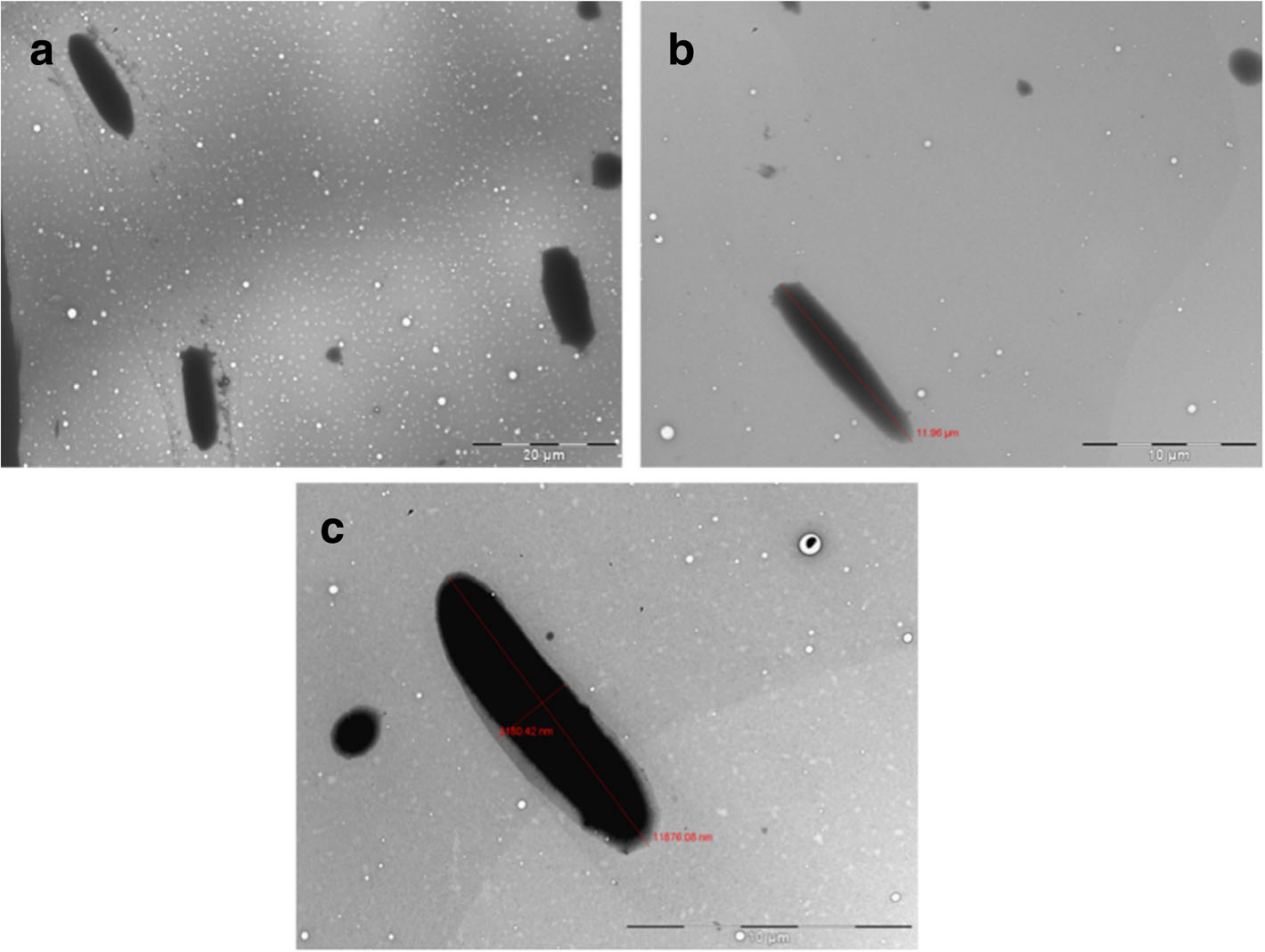
**a-c**: The PCM of a single cell of *M. smegmatis* observed under TEM catapulted into 1% PTA from glass slides and compared to a cell grown in nutrient media. **a** Several catapulted individual cells **b** The intact single cell successfully catapulted and isolated using LPC **c** *M. smegmatis* (NCIMB8548) cell isolated from the nutrient broth (48 h, 37 °C). Scale bar represents 20µm in panel a and 10µm in panels b and c

In this study, the ZN stained slides were examined under the LCM to identify an isolated or “not over crowded” area. The area containing isolated cells was circumcised and the appropriate laser energy was applied to catapult individual cells. Lower LCM laser energy (below 80%) is recommended by the manufacturer to avoid cell damage. However, we indicated that by utilizing laser energy between 80 to 85% is the most appropriate for isolation of cells stained and fixed to archived slides. The quantification of PCMs was performed to determine the sensitivity of the system for detecting the lower number of cells catapulted by LCM. This technique was performed in order to obtain as many individual cells as possible. Each successful catapulting process was determined by rechecking the catapulted area under 63 × magnifications and recording the blast numbers. In order to ensure that the PCMs were being successfully catapulted, we re-examined the PCMs at 63× magnification of LCM. 5 μl of each sample was transferred onto a clean glass slide and re-examined at the same magnification with no additional staining for the presence of PCMs (Fig. 1c). This is the first study to show that the LCM can be utilised to isolate intact bacteria cells from ZN stained archived glass slides (Fig. 2 a-b). Significantly, when using an appropriate percentage of laser energy, LCM does not negatively impact the morphology of the catapulted bacteria allowing individual rod-shaped cells (~ 3.12 μm by 12.8 μm in size) to be observed under the TEM. The size and shape of the catapulted cells was compared to the *M. smegmatis* cells grown in the nutrient broth media for confirmation (Fig. 2 a-b). These results verify the ability of this technique to obtain single bacterial cells fixed on glass slides without significantly affecting their morphology. Given that contaminants such as other bacterial cells or dust particles attached to the slide surface could be catapulted together with the cells of interest, this approach can also be used to identify if targeted cells had been recovered and eliminates other contaminants. By catapulting intact individual cells, the probability of capturing DNA for genomic DNA extraction is significantly increased. However, given that ZN staining is likely to damage the genomic DNA, further investigation is needed to check the integrity of the nucleic acid. Scale bar represents 10µm in panels a-c.

### Determination of the minimum number of cells detected using the combined multiple displacement amplification MDA-nested PCR system

This study was conducted to identify a suitable method to extract adequate amounts of DNA for amplification from very low numbers of *M. smegmatis* cells, catapulted from archived glass slides by LCM. Three extraction methods chosen for this study included heat-shock, heat shock-DNA precipitation and QIAamp DNA Micro kit. In each case, DNA extraction was carried out using 5, 10, 15, 20, 25 and 30 of catapulted cells. All the extraction methods chosen, showed similar DNA recovery based on concentrations determined by the NanoDrop 2000 spectrophotometer (Thermo Scientific, USA). The genomic DNA extracted using all three different methods was used as a template for the pre-amplification step using the MDA kit. This pre-amplification step provided increased amounts of DNA template for primary PCR (Table 1). Amplicons obtained from the primary PCR were subjected to a pre-wash step using Millipore Amicon®Ultra-0.5 ml centrifugal filter devices (Millipore, USA). This removed external primers and inhibitors before employing the nested real-time PCR. As a result, a primary amplicon obtained from the genomic DNA from 30 post catapulted cells gave a detection signal. There were no amplifications observed from other catapulted cells. Amplifications were consistently observed using a minimum of 30 catapulted cells for genomic DNA extraction.

**Table 1:** The concentration and purity of DNA extracted using different methods and followed by MDA using REPLI-g® UltraFast Mini kit

Samples	DNA extraction	Pre-MDA		Post-MDA			
				Undiluted		1:25 dilution in ddH_2_O	
		Purity (260/280 ratio)	Concentration (ng/μl)	Purity (260/280 ratio)	Concentration (ng/μl)	Purity (260/280 ratio)	Concentration (ng/μl)
LCM 1	Heat shock	1.52	4.5	1.81	666.1	1.69	31.6
LCM 2	Heat shock followed by ethanol precipitation	1.55	2.3	1.80	730.4	1.65	52.3
LCM 3	QiaAmp DNA Micro kit (Qiagen)	1.58	3.6	1.81	765.1	1.60	32.5
Positive control	Heat-shock from single colony	1.61	72.3	1.89	876.4	1.68	46.8

Two sets of *rpoB* gene-specific primers used in this study were optimized using *Mycobacterium smegmatis* (NCIMB 8548) and an environmental isolate of *M. smegmatis* (VS/02 generously provided by Dr. Nigel Ternan, Ulster University) as a positive control. In addition, the primers were also optimized and validated using commercially available *Mycobacterium tuberculosis* DNA (ATCC®25177) which ensured that they detect different, clinically relevant, *Mycobacterium* species (results not shown).

**Fig. 3:**
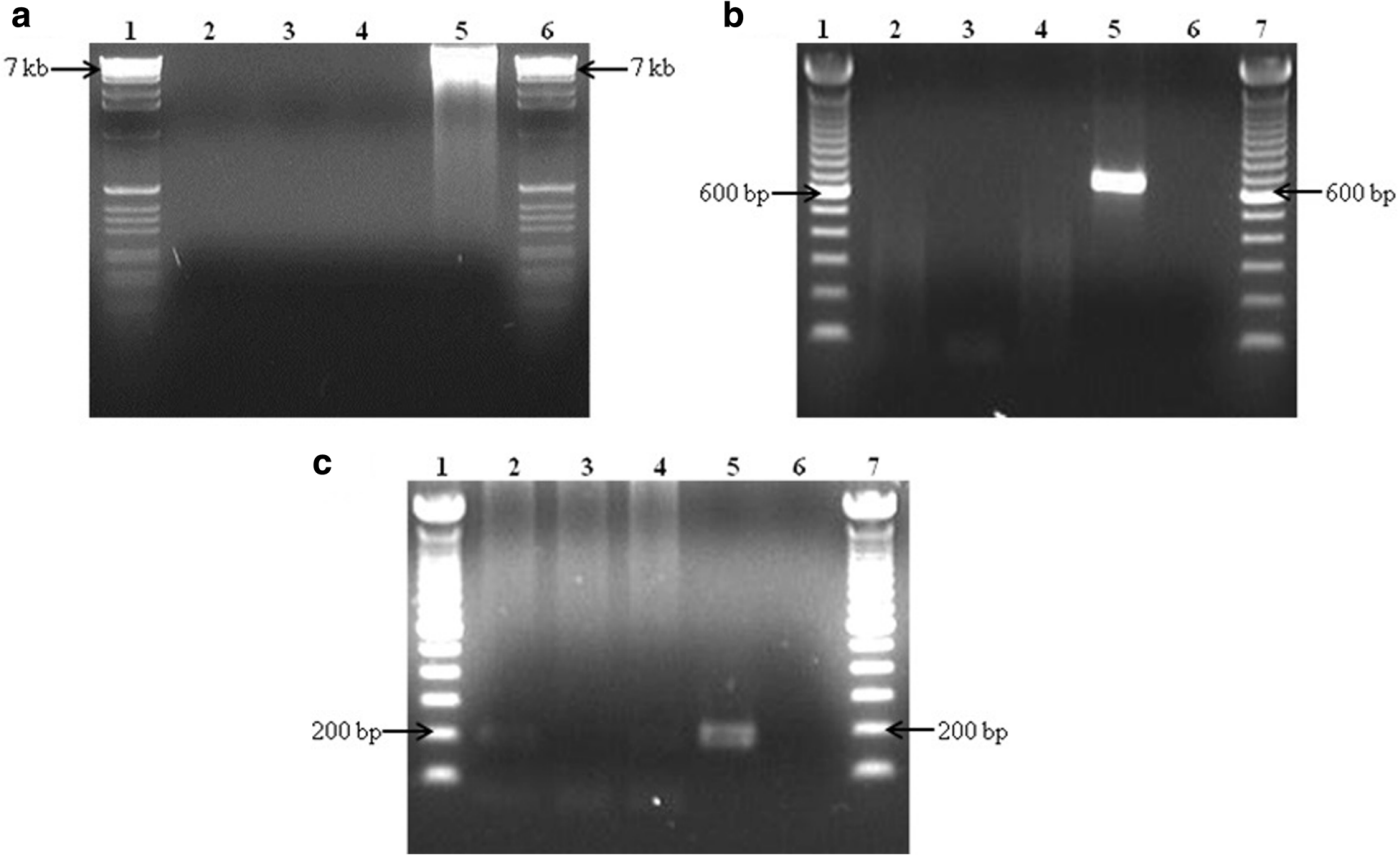
(i, ii and iii): Agarose gel electrophoresis (1.5%, 4 °C) showing the amplification process of 30 *M. smegmatis* (NCIMB 8548) cells catapulted from Ziehl-Neelsen archived slide using LCM. (i) Multiple displacement amplification (MDA) by using REPLI-g® UltraFast Mini kit (Qiagen). Samples were extracted using 3 different methods to determine an appropriate method for a lower number of cells isolated from archived glass slides. Lane 1 and 6: 100 bp ladder; Lane 2: Heat-shock; Lane 3: Heat-shock followed by ethanol precipitation; Lane 4: QIAamp DNA Micro kit; Lane 5: positive control. (ii) 600 bp product of primary PCR using post-MDA mixture as a template which was performed by using touchdown PCR. Lane 1 and 7: 100 bp ladders; Lane 2: Heat-shock; Lane 3: Heat-shock followed by ethanol precipitation; Lane 4: QIAamp DNA Micro kit; Lane 5: positive control; Lane 6: negative control. (iii) 176 bp product of touchdown nested PCR amplified from primary amplicon. Lane 1 and 7: The 100 bp ladders; Lane 2: Heat-shock; Lane 3: Heat-shock followed by ethanol precipitation; Lane 4: QIAamp DNA Micro kit; Lane 5: positive control; Lane 6: negative control

Samples were amplified using the MDA kit without primers and the products were analyzed using agarose gel electrophoresis. Amplification was observed only for the positive control (Fig. 3 (i)). Despite the lack of sample amplification, the MDA reactions were used as templates for the primary PCR. However, the primary PCR of the samples did not show the expected 600 bp product as observed for the positive control (Fig. 3 (ii)). These results suggest that the sample amplicons generated were insufficient for detection on an agarose gel after a single round of PCR.

A nested touchdown PCR was also used to amplify the *rpoB* gene. Comparing the sensitivities of normal PCR and real-time PCR using the first round PCR products as templates revealed that the nested real-time PCR increased the sensitivity by 30-fold (Figs. 3 (iii) and 4). In separate study, in which the MDA reaction step was omitted, we were able to detect a signal for the nested real-time PCR using approximately 100 catapulted cells per slide as opposed to 5,000 cells for normal nested PCR for the *rpoB* gene (data not shown). The 5-fold diluted primary PCR amplicon when used as a template for the nested real-time PCR exhibited a delayed quantification cycle (C_q_) value in comparison to the undiluted primary PCR amplicon (data not shown). The desired amplification product could be not be detected on a 1.5% agarose gel when the diluted primary PCR amplicon was used a template for a nested PCR.

**Fig. 4:**
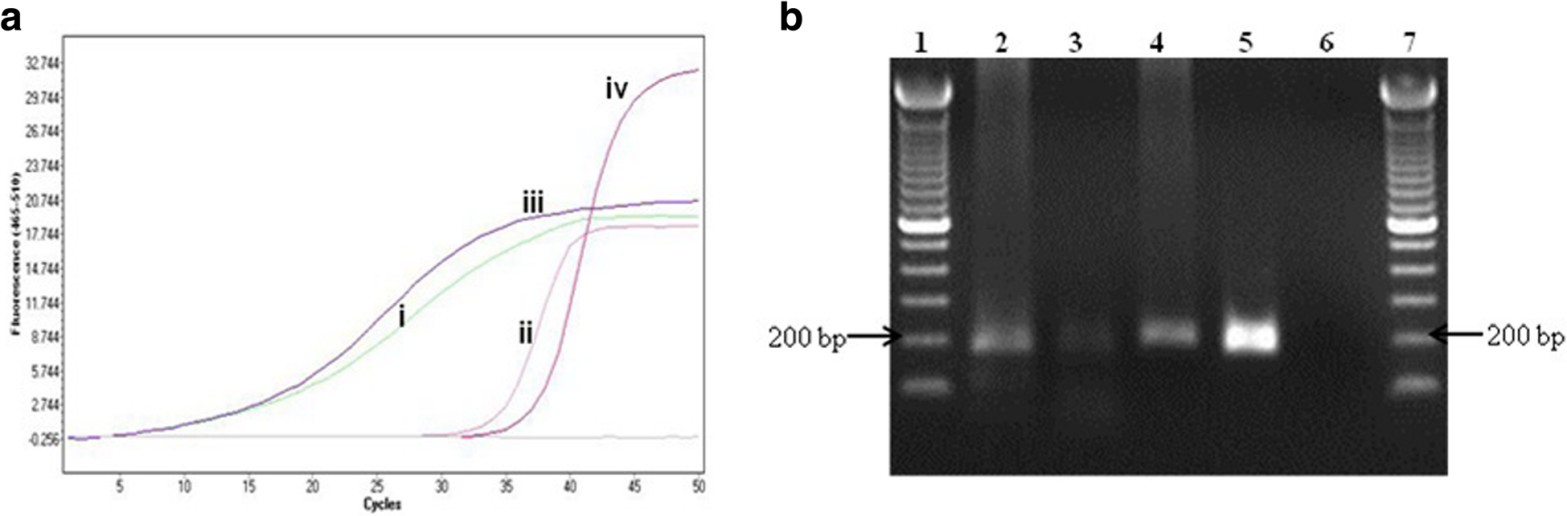
**a** and **b**: Nested real-time PCR of the primary PCR amplicon amplified from pre-processing 30 catapulted cells from a glass slide using LCM. **a** Quantification cycles show early detection of undiluted primary amplicon compared to the positive control. The positive control was diluted at 10-fold to avoid primer saturation. **b** Agarose (1.5%, 4 °C) gel electrophoresis showing the 176 bp nested real-time PCR amplification. The amplification was performed by using primers BnMS949bf and 1105br. Lane 1 and 7: 100 bp ladder; Lane 2: Heat-shock; Lane 3: Heat-shock followed by ethanol precipitation; Lane 4: QIAamp DNA Micro kit; Lane 5: positive control; Lane 6: negative control

## Discussion

Diverse methods to identify *Mycobacterium sp.* (commonly for *Mycobacterium tuberculosis* complex (MTBC)) have emerged with significant improvements in PCR technology. Researchers have developed accurate, rapid, reliable and cost-effective methods to fulfil demands of the diagnostic laboratories [23]. The use of fresh clinical specimens is crucial in many of the molecular-diagnostic based applications. In most instances, clinical specimens such as sputum must be within 48 h old prior to processing to ensure cell reactivity [24–27]. Many diagnostic studies have also been developed using freshly cultured bacteria [28]. In spite of the advantages to using fresh samples, several studies have reported that *Mycobacterium* sp. have been detected from archived or long-storage clinical specimens [29, 30]. There are some challenges faced for archived samples so an adequate amount of amplifiable DNA can be obtained. This limitation has prompted studies to use stored and archived samples for molecular detection. Multiple displacement amplification (MDA) which utilises Φ DNA polymerase has been reported to be a highly effective application for archived and small amount of samples [21, 22, 31, 32].

Based on these previous studies, we developed a molecular detection system which involves using *M. smegmatis* as a model organism to obtain confirmatory information from sputum stained slides. This involved optimization of LCM as a useful isolation tool for bacterial cells and molecular detection of a very low number of isolated cells involving three steps: a pre-amplification step using MDA, primary touchdown PCR and nested real-time PCR. Our study differs from previous studies as molecular detection has been performed at a single cell level instead of infected tissues/membrane. It was possible to detect DNA from low numbers of *M. smegmatis* that were stained using a standard Ziehl-Neelsen protocol. We showed that MDA as the pre-PCR step greatly enhanced molecular detection when the amount of starting material was relatively low. LCM was an efficient tool for isolating individual bacteria cells which were heat-fixed and stained. We have also demonstrated that the staining procedure did not have an effect on the overall detection system.

While a number of previous studies have focused on developing rapid detection methods for *Mycobacterium* sp*.* from materials fixed on glass slides, such as sputum samples [8–10], none have used LCM as an isolation tool, instead employing techniques such as scraping to maximize bacterial cell recovery. Studies have shown that these latter approaches can lead to carryover of adjacent tissues (possibly from sputum or microdissected tissues) which can reduce the PCR detection efficiency [33–36].

In most instances, low numbers of cells are present in sputum smears, which are usually difficult to detect and it is at times difficult to differentiate between stain debris and stained bacilli which could be easily misinterpreted by an inexperienced observer [37]. The development of a system that could identify the presence of these bacilli could be beneficial in diagnosing tuberculosis. LCM has the same function as a normal light microscope and can be used to detect the presence of bacteria from clinical specimens. Indeed, several studies have previously demonstrated the potential of LCM for removing bacterial targeted areas from infected cells [38, 39]. It has also been shown that LCM minimizes contamination from adjacent or non-required cells or tissue, reducing inhibitory effects on subsequent PCR amplification steps. [40, 41]. Another significant advantage of the system is the speed of diagnosis; molecular detection reduces diagnostic turnaround times from 12 weeks (for culture based techniques) to a matter of hours for molecular diagnostics [42]. While the initial costs of establishing the system are likely to be prohibitive, especially in less developed countries with the high numbers of tuberculosis cases, this limitation can be overcome by simply mailing the slides to a central facility, either nationally or internationally. The fixed slides pose minimum biological risk compared to fresh clinical specimens such as expectorant [10, 37]. Furthermore, irrespective of capital costs, LCM has an extremely low unit cost per sample, making it a competitive diagnostic technology application [43]. Molecular diagnostic results can then be sent back to the clinician by email or text, facilitating a much quicker turnaround than would be required for in situ culture based detection.

In this study, we used LCM to catapult, quantify and re-examine the catapulted cells to ensure that they had totally detached from the surface of the slides. We were able to show the catapulted cells remain intact even after the application of appropriate laser energy. The presence of whole intact cells in the catapulted material increased the chances for extracting “inbound” or non-denatured DNA.

In current clinical practice, the carbol-fuchsin stained smears were prepared for the confirmation of positive or negative of the presence of acid-fast bacilli (AFB). It is observed thoroughly based on the 300 fields of microscopy observation [44]. Until now, no accurate methods have been used to quantify the number of bacteria cells fixed on glass slides. The samples collected by using LCM often produce low numbers of PCMs; therefore it is crucial to select the appropriate DNA extraction method for obtaining an adequate amount of genomic DNA. While the commercial kit available for LCM samples showed consistency in producing enough DNA for amplification, the heat-shock method can also be used as an alternative for efficient extraction of genomic DNA from PCMs. This method is particularly useful when working with a large number of samples. Any extraction method involving cell lysis can be used for mycobacteria as this genus comprises a thick cell wall [45]. Cell lysis was a crucial step in obtaining adequate amounts of non-denatured DNA. The heat-shock method was the most rapid and effective method for obtaining genomic DNA from a low number (< 100) of catapulted cells. Another study in our laboratory has shown that the heat-shock method resulted in inconsistent amplification when used for more than 100 catapulted cells. These results suggest that using more than 100 catapulted cells for the heat shock method may result in the presence of inhibitory factors which may interfere with further processing of the sample. Amplification using DNA extracted from a low amount of cells (0.05 μl blood) can eliminate interference due to the presence of inhibitory factors [46]. It has been supported by other studies which involve human clinical specimens [13] and freshly cultured bacteria [18, 20–22, 31, 32, 47]. The selection of method for DNA extraction for PCMs can be determined based on the amount or sample volume. Inconsistencies observed with the heat shock-ethanol precipitation method for extracting DNA suggest that the precipitation step is not essential in order to obtain adequate amounts of DNA. This additional step increases the purity of the DNA but any residual ethanol could easily inhibit the amplification process [48, 49].

Processing low numbers of catapulted cells can be a challenge. We demonstrated the application of MDA to increase the yield of the template for the primary PCR for specific detection of PCMs isolated from glass slides. This technique has recently been used for the detection of single bacterial cells [50]. Previous studies have reported that the MDA technique is extremely effective for amplifying DNA extracted from traces or very low amounts of sample, especially in forensic work [15, 16]. This technique has also been used for detection of bacteria from various sources such as environmental [17], clinical [31, 46] and agricultural specimens [18]. Recent studies have also shown the reliability of MDA as an additional step for increasing the yield of DNA template for the primary PCR from slow growing *Mycobacterium* sp. [13, 32]. However, a significant advantage of our system, over other diagnostic approaches including GeneXpert [51], is that it allows highly discriminatory single cell detection from archival material, including samples which have been stained and fixed to glass slides. Indeed, while others have used LCM followed by molecular diagnosis of *Mycobacterium* from fresh tissue samples [39, 52], we are the first to do so successfully from archival materials; potentially facilitating large scale retrospective epidemiological studies.

Studies have shown that amplification was possible using as little as 1 ng DNA from 300 nucleated cells [19]. We show that the amplification of genomic DNA from catapulted cells was visualized on the agarose gel after nested PCR was performed. This finding suggests that the primary amplification likely occurs, albeit not visible on an agarose gel. This also indicates that the amount of starting materials (in this case number of catapulted cells) does have an effect on the amplification in order to obtain adequate amplifiable DNA. Our observations indicate that a single PCR could not be amplifying a low number of catapulted cells from slides. Our optimization study has shown detection using nested real-time PCR increased sensitivity by 30-fold compared to the normal nested PCR. Additionally, the sensitivity of detection decreased by 5 and 10-fold respectively when 10 and 100-fold had been applied, based on the quantification cycle (Cq) (> 36 cycles) and also that no amplification product is observed on the agarose gel analysis (data not shown).

It has been reported that if the genomic DNA is purified prior to amplification the possibility exists for both DNA template loss and damage to occur. Therefore we designed our MDA system to work directly on gently lysed cells to prevent these complications and reduce amplification bias that could result from degraded DNA templates [14]. Of even greater significance is the use of a high-fidelity proofreading DNA polymerase in the MDA process. Taq DNA polymerase has an error rate of ~ 3 in 10^4^ with an estimated accumulation of one mutation per 900 bases after 20 PCR cycles. The Φ DNA polymerase has a significantly reduced error rate of 1 in 10^6^–10^7^ nucleotides and consequently the mutation rate following a 10,000-fold amplification by Φ DNA polymerase is only around 3 per 10^6^ nucleotides [53]. This will allow for increased accuracy in applying sequence based molecular epidemiology tools on the material recovered from archived slides.

In summary, the system described herein can be used for microbial epidemiology studies as well as serving as a platform for rapid diagnosis of pathogens, especially those which are difficult to culture. We initially developed the system with a view to applications in *Mycobacterium* diagnosis and epidemiology but there are many potential applications in other diseases that rely heavily on microscopy for diagnosis. One such disease is mycetoma, a neglected tropical disease urgently requires improved pathogen identification in order to inform patient management and predict disease treatment and outcomes. Regardless of gene specific targets or amplification methods, the MDA system provides templates from a minimum of 30 isolated bacteria from the glass slides. In line with the development of many microbial detection techniques, this system can be combined with other detection applications such as microarray and MALDI-TOF in order to obtain rapid and reliable data. Time-consuming detection systems could potentially delay appropriate treatment for TB cases. Thus, the development of a molecular detection system involving samples recovered from glass slides could serve as a platform for rapid diagnosis of *Mycobacterium* sp.

## Conclusions

To our knowledge this is the first time that the technique of MDA has been applied for single cell detection from archival material i.e. samples stained and fixed to glass slides, as opposed to fresh tissue. We feel that this approach has significant potential, not only as a useful diagnostic platform for prospective studies, but also opens the possibility for retrospective epidemiological studies from archival material.

## Methods

### *Mycobacterium smegmatis* culture and Ziehl-Neelsen (ZN) stained slide preparation

Lyophilized *Mycobacterium smegmatis* (NCIMB 8548) was re-hydrated with 0.5 ml double-distilled water (ddH_2_0) and sub-cultured on Nutrient agar and Brain Heart Infusion (BHI) broth (both from Sigma-Aldrich, UK) at 37 °C for 24 to 48 h. The strain used for preparing the slides had previously tested positive for confirmation using a set of *rpoB* gene specific primers (BMS738aF/BMS1311aR). Primer details are listed in Table 2. Smears were prepared by spreading colonies isolated from an agar plate onto 1 mm thick glass slides. Subsequently, the traditional Ziehl-Neelsen hot method was carried out using the standard protocol [54] and observed under a normal light microscope for the presence of *M. smegmatis*. All slides were kept at room temperature for a minimum of three months prior to use for archiving.

**Table 2:** List of primers used in this study and for detection of *Mycobacterium smegmatis* (NCIMB 8548), *M. smegmatis* environmental isolate (VS/02) and genomic DNA from *Mycobacterium tuberculosis* (ATCC®25177).

Primer	Primer sequences (5′-3′)	Gene	Location	Annealing temperature (°C)	Sequence reference (GenBank accession number)	Product sizes (bp)	References
BMS 738aF	GAC AAG TCC ACC GAG AAG AC	*rpoB*	738–758	59	AY262735 U24494	593	Present study
BMS 1311aR	ATC TGG TTC TGG ATC AGC TC	*rpoB*	1311–1331				Present study
BnMS 949bf	TGG AGA AGG ACA CCA CCT	*rpoB*	949–967	59	AY262735 U24494	179	Present study
BnMS 1105br	CAG CTT CTT GTT GAC CTT GT	*rpoB*	1105–1125				Present study

### Observation and isolation of samples fixed on glass slides using LCM

The PALM (Microlaser, Bernried, Germany) laser microdissection system was used to detect and dissect single and multiple cells of *M. smegmatis* from archived slides as described by with modification [43]. In order to obtain the minimal number of catapulted cells, samples were extracted from isolated or low-density regions of the bacterial smears, followed by catapulting with the aid of a laser pulse. The laser pulses were maintained between 80 to 85% to ensure that the post-catapult materials (PCMs) remained intact. The catapulted cells were collected in caps of 0.5 ml microcentrifuge tubes inverted above the sample slide by using the laser pressure catapulting (LPC) application of LCM. For sensitivity tests, triplicate samples were obtained by individually catapulting samples, ranging from 10 to 30 cells, into microcentrifuge tube caps containing 30 μl ddH_2_O. The catapulted samples were collected by inversion and centrifuged briefly for storage at 4 °C until further analysis.

### Observation of the PCMs under transmission electron microscope (TEM)

The Tecnai™ Transmission Electron Microscope 12 spirit 120 kV (FEI, The Netherlands) was used to view the post-catapult materials recovered from glass slides by using laser capture microscope (LCM). The Formvar-coated grid disc and TEM for this study was generously provided and utilized by Dr. Barry O’Hagan (UUC). Two types of samples were prepared by using a standard protocol of TEM sample preparation with slight modification for post-LCM application.

### Sample preparation for TEM from fresh culture

In order to obtain morphological information on *M. smegmatis*, a fresh culture of cells was prepared by growing the *M. smegmatis* in nutrient media for 48 h and incubating at 37 °C. Cells were then harvested by transferring 50 μl culture suspensions into clean 0.5 ml microcentrifuge tubes and centrifuging at 3000×g for 10 min. Subsequently, the supernatant was discarded and pelleted cells were washed and resuspended with ddH_2_O centrifuged at 10,000×g for 10 min. This step was repeated three times to remove any contaminants from growth media. From the washed samples, 5 μl was mixed with 5 μl 1% phosphotungstic acid (PTA) (Sigma-Aldrich, Germany) in a clean 0.5 ml microcentrifuge tube and left at room temperature for 2 min. The mixture was vortexed and centrifuged briefly before transferring a drop to a clean glass slide. Immediately, a Formvar-coated grid disc was placed on top of the droplet and allowed to stand for 2 min. The Formvar-coated grid disc was turned upright and air-dried before visualizing under the TEM.

### Sample preparation from the PCMs

Catapulted materials were obtained from archived slides by using the LPC application of LCM. To observe the PCMs under TEM, the *M. smegmatis* cells were catapulted directly into the 1% PTA. From this 10 μl of 1% PTA was aliquoted into a 0.5 ml clean microcentrifuge cap and the same procedures from LCM application were performed, as described in section 2.3.6. The cap was carefully removed from the PALM® RoboMover and the Formvar-coated grid disc was immediately placed on the suspension for exactly 2 min. This was air-dried prior to visualizing under the TEM.

### The DNA extraction

In order to determine the most suitable method to extract DNA from low numbers of cells catapulted using LCM, three different DNA extraction methods were performed. Unless otherwise stated, all centrifugation steps were performed using a 5417R refrigerated microcentrifuge, 115 V equipped with fixed angle rotor (Eppendorf, UK). All PCM was subjected to one of the three DNA extraction methods without any treatment. Prior to being subjected to MDA, the concentration and purity of the extracted DNA was determined using a NanoDrop 2000 spectrophotometer (Thermo Scientific, USA).

#### Heat-shock

The DNA was extracted from the PCM by using a heat shock method. The PCM samples (30 μl), previously catapulted by LCM from the glass slides, were incubated at 95 °C for 10 min using a heat block (Grant Instruments, England). Subsequently, the suspension was centrifuged immediately at 13,000×g for 10 min to pellet debris. The supernatant containing the DNA was transferred to a clean microcentrifuge tube and kept at -20 °C for future use.

#### Heat-shock-ethanol precipitation

Ethanol precipitation was performed following the heat shock method. A batch of heat-shocked prepared samples were subjected to ethanol precipitation by a slightly modified method [48]. Briefly, 1/10 volume of 3 M sodium acetate solution (pH 5.2) (Fermentas, UK) was added to 30 μl of dissolved DNA, followed by the addition of 1 μl of 1µg/μl glycogen (Fermentas, UK) per 20 μl of solution and gently mixed. The mixture was incubated for 5 min at room temperature and immediately centrifuged for 15 min at 10,000×g at 4 °C. The pellet was rinsed with cold 70% ethanol and air-dried. The pellet was dissolved in 50 μl nuclease free water and kept at -20 °C.

#### QIAamp DNA micro kit (Qiagen, USA)

The third DNA extraction method utilized a commercial DNA extraction kit specifically for microdissected tissues isolated using laser capture microscopy - the QIAamp DNA Micro kit. A 30 μl sample that contained different numbers of cells was centrifuged at 13,000×g for 10 min and 10 μl of water was removed. The DNA extraction was performed in accordance with the manufacturer’s instructions by using 20 μl volumes from the starting materials.

### Pre-amplification step using MDA

MDA was performed using a protocol for purified genomic DNA provided by Repli-g® UltraFast Mini kit (Qiagen, Germany) as described by the manufacturer with some modification. Post-MDA mixture was diluted to 1:25 in ddH_2_O in accordance with the manufacturer’s instructions, however there were a set of samples also prepared without dilution prior to being subject to molecular detection. The concentration of samples was determined by using NanoDrop 2000 spectrophotometer (Thermo Scientific, USA).

### Primary and nested touchdown polymerase chain reaction (PCR)

Both primary and nested PCR were performed in a final volume of 50 μl containing 1× of PCR buffer (50 mM Tris-HCl; 75 mM KCl; 10 mM MgCl_2_), 2.5 unit of Taq polymerase, 1.5 mM of 50 mM MgCl_2_, 2 mM of each 100 mM dNTP, 20 pmol/μl forward and reverse primers and 1 μg of DNA samples. All chemicals used were obtained from Invitrogen, Inc. (UK). The touchdown PCR was carried out on a Techne® TC-5000 Thermocycler (Cole-Parmer, UK) using the following cycling conditions: initial denaturation for 4 min at 95 °C, 5 cycles of denaturation at 95 °C, primer annealing for 1 min at 60-56 °C (1 °C decline for each cycle) and extension at 72 °C for 1 min, followed by a further 30 cycles at denaturation for 5 min at 95 °C, annealing at 55 °C for 1 min, extension at 72 °C for 1 min and finally amplification is ended with a extension at 72 °C for 5 min.

### Pre-washing for inhibitory removal

In order to ensure no contaminants or inhibitors were carried over to the secondary PCR (nested PCR), Millipore Amicon®Ultra-0.5 ml centrifugal filter devices (Millipore, USA) were used to remove primers from the primary amplification as per the manufacturer’s protocol with modification. Briefly, 30 μl of primary PCR amplicon and 30 μl of 1× Tris-EDTA (TE) buffer were transferred into the combined devices (filter and collection tubes) and centrifuged at 14,000×g for 10 min. This procedure was repeated twice with the second volume of 1× TE buffer reduced to 20 μl. The filtered samples were used as the template for secondary PCR.

### Nested real-time PCR

Nested real-time PCR was performed using the pre-washed amplicon previously amplified using primary touchdown PCR. The amplicon was re-amplified using the internal primer pair BnMS949bf and 1105br which produced amplicons of 176 bp. All real-time PCR equipment and reagents were obtained from Roche Diagnostics GmbH, Mannheim, Germany and Roche Diagnostics, Hertfordshire, United Kingdom. Real-time PCR was performed with LC480 LightCycler using LightCycler® 480 SYBR Green I Master, which was used in accordance with the manufacturer’s instructions. The amplification was performed under the following condition: initial denaturation of 10 min at 95 °C which was followed by 45 cycles of 15 s of denaturation at 95 °C, 30 s of annealing at 58 °C and 60 s of extension at 72 °C. These protocols had been optimized by using the samples isolated from the glass slides by the scraping method.

### Agarose gel electrophoresis

The PCR products were analysed with agarose gel electrophoresis. A mixture of 10 μl of PCR product with 2 μl of 6× loading dye was resolved in a 4 °C 1.5% agarose gel in 1× Tris-Borate-EDTA (TBE) buffer. Electrophoresis was performed horizontally and submerged in 1× TBE buffer. The PCR products were separated at 80 V for 1 h and visualized using ethidium bromide (0.5 μg/ml) staining. The gel was observed for the presence of the products under a combined unit of UV-light transilluminator and image photographing unit using AlphaImager™ 2200 (Alpha Innotech, USA).

## Abbreviations

BHI: Brain Heart Infusion
BP: Base pair
LCM: Laser Capture Microscopy
LPC: Laser pressure catapulting
MALDI-TOF: Matrix Associated Laser Desorption Ionisation-Time of Flight
MDA: Multiple Displacement Amplification
MDR-TB: Multi-drug resistant TB
NTM: Non-tuberculous mycobacteria
PCM: Post-catapult materials
PCR: Polymerase Chain Reaction
PTA: Phosphotungstic acid
TB: Tuberculosis
TBE: Tris Borate EDTA
TE: Tris EDTA
TEM: Transmission Electron Microscopy
WGA: Whole genome amplification
ZN: Ziehl-Neelsen
